# Additional challenges faced by cancer patients in Gaza due to COVID-19

**DOI:** 10.3332/ecancer.2020.ed100

**Published:** 2020-04-22

**Authors:** Shaymaa AlWaheidi, Richard Sullivan, Elizabeth A Davies

**Affiliations:** 1 Cancer Epidemiology, Population and Global Health, King's College London, London, UK; 2 Institute of Cancer Policy, Kings Health Partners Comprehensive Cancer Centre, King's College London, London, UK

**Keywords:** COVID-19, breast cancer, Gaza

## Abstract

Cancer patients in conflict settings experience significant barriers in accessing chemotherapy and radiotherapy as well as palliative care and psychosocial support. Now they face an additional possible risk of infection by SARS-CoV-2 novel coronavirus and the indirect impact of the COVID-19 pandemic on movement restrictions and their access to care. In this commentary, we highlight that despite the low COVID-19 burden in conflict settings like Gaza, COVID-19 could lead to further inequity in cancer care and poorer outcomes for Palestinians with cancer. This is due to the pre-existing shortage in cancer resources as well as the lack of context-specific guidelines to prepare for COVID-19 in war-torn settings.

Under ‘normal’ circumstances, cancer patients in Gaza, a conflict setting within the occupied Palestinian territory and home for more than 1 million refugees, have limited access to many cancer treatments. This requires them to wait for many weeks before they receive Israeli travel permits to leave Gaza and access treatment [1]. Prior to the COVID-19 pandemic in February 2020, 69% of 1760 patients applying for a medical exit permit received one in time for their appointment, 9% were denied and 22% were delayed access, receiving no response to their application by their treatment appointment date [2]. Once the COVID-19 pandemic started in late February/early March in Gaza, there was a substantial tightening of restrictions on movement. To prevent the spread of COVID-19, both the Israeli authorities, and the Palestinian authorities in Gaza and the West Bank now request that patients do not leave Gaza, except for in emergencies [3]. There are many countries implementing extreme social distancing measures—lockdowns—however, there is not yet evidence of how the pandemic response has impacted cancer patients.

Prior to the introduction of COVID-19 in Gaza, we conducted semi-structured qualitative interviews with 20 women diagnosed with breast cancer between 2017 and 2018. Following the start of COVID-19 in early March 2020, we followed up these women to examine changes in their health and experiences of care. We found new concerns emerging about the impact of COVID-19 on their treatment. For example, Zeinab (not her real name), aged 30, had been receiving the hormonal blocker Goserelin (not available in Gaza) for several months at the Augusta Victoria Hospital, a non-governmental hospital in East Jerusalem. This month, Zeinab revealed she has cancelled her appointments in Jerusalem because she fears that upon returning to Gaza she would be sent to a quarantine centre for 21 days—and kept further away from her children. Zeinab would not have had to make such a decision had Goserelin or testing been available in Gaza. Another patient, Marwa (not her real name), aged 45, has decided to stay in Jerusalem for her next round of radiotherapy—thus lengthening her separation from her family. Radiotherapy is also not available in Gaza and the West Bank, so patients need to travel to receive it at the Augusta Victoria Hospital. Marwa said ‘I was lucky to receive an exit permit to access radiotherapy in Jerusalem, and I do not want to go back to Gaza in case more cases are discovered and they do not allow me another travel permit to continue my treatment here’.

On top of that, a number of women expressed concerns about catching COVID-19 in the hospital setting, especially given that current recommendations on social distancing do not seem to work well at the two oncology departments in Gaza. On a normal day up to 150 cancer patients, and their family members, cram into the oncology department at Rantisi Hospital, where there is just one toilet facility for everyone to use. In addition, every 4-6 cancer inpatients share a single room and one toilet. This close unprotected proximity increases contact between patients, and increases the risk of catching COVID-19 from the hospital visitors.

As of 5 April, a total of 1301 samples have been tested and 12 cases of COVID-19 have been confirmed in Gaza (Figure 1) [4]. The low number of cases in Gaza and elsewhere in the occupied Palestinian territory are likely to reflect the lack of testing. It is this bottleneck that made the Palestinian government extend the mandatory self-isolation for people in quarantine sites to 21 days from 14 days [3].

Being a demographically young population (4% are aged 65 years or over and have the greatest prevalence of pulmonary and cardiovascular diseases and diabetes), most COVID-19 patients will recover in Gaza. However, those who develop serious respiratory distress, mainly but not exclusively those with comorbidities (36% of people above 50 in Gaza have diabetes) [5] will need invasive mechanical ventilation. Only 63 mechanically ventilated beds are currently available in governmental hospitals [4], with more than two-thirds already in use by other patients). Therefore, those who need more invasive support, which is mostly not available, will likely die.

The World Health Organisation (WHO) and other international health organisations have already raised the alarm about the significant shortage of Personal Protective Equipment (PPE) (until March 31, only 2,500 were available) [6] and the deficit in drugs and power supply in Gaza. However, the long-term consequences of COVID-19 on high income economies means that overseas aid is likely to be slashed. Meanwhile, there are ongoing efforts by the Palestinian Ministry of Health and WHO to transform a hospital in southern Gaza into a hub for possible COVID-19 patients [6]. However, the hospital currently provides care for about 30% of all cancer patients in Gaza and there is thus uncertainty as to how this is going to affect provision of cancer care for such patients.

## Conclusion

COVID-19 both directly and indirectly is exacerbating inequity in cancer care and will lead to further poorer outcomes for Palestinians with cancer. It is also compromising the quality of life of cancer patients in a catastrophic socio-political context where the suffering of patients is already compounded by the acute shortage of diagnostic and treatment facilities as well as the lack of trained cancer specialists.

## Conflicts of interest

We declare no competing interests

## Funding declaration

This publication is funded through the UK Research and Innovation GCRF RESEARCH FOR HEALTH IN CONFLICT (R4HC-MENA); developing capability, partnerships and research in the Middle and Near East (MENA) ES/P010962/1.

## Figures and Tables

**Figure 1.**
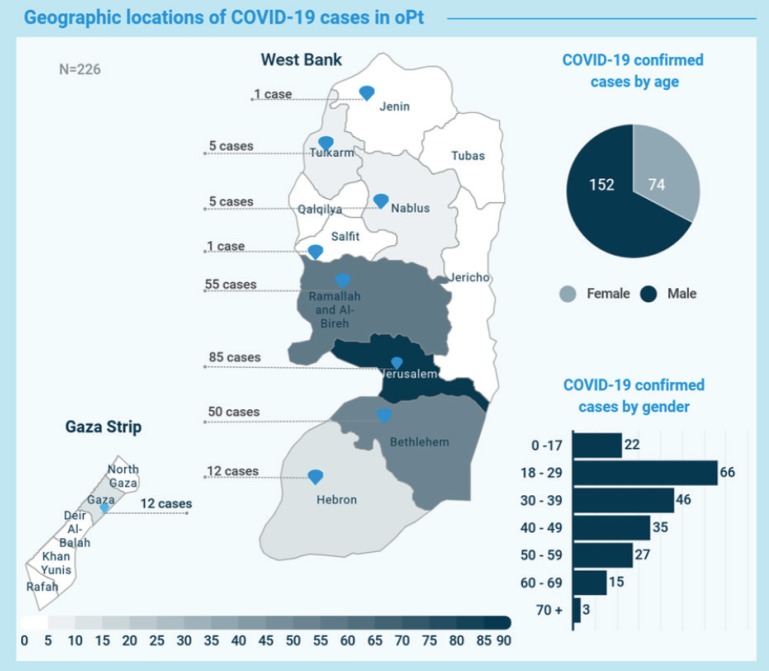
WHO Coronavirus disease (COVID-19) oPt Update 19 (April 5, 2020) [4].
